# Exploring Afghan Refugees’ Post-Resettlement Experiences in North America: A Scoping Review

**DOI:** 10.1177/08445621261420300

**Published:** 2026-02-11

**Authors:** Hasina Amanzai, Betty Q. Wang, Cristina Catallo, Sepali Guruge, Souraya Sidani, Bharati Sethi, Erin Ziegler, Stephanie Nishi, Pheba Joy, Angelina Stafford, Andrea Borges, Mushgan Sediq

**Affiliations:** 1 Daphne Cockwell School of Nursing, Faculty of Community Services, 7984 Toronto Metropolitan University, Toronto, ON, Canada; 2 School of Nursing, 56014 York University, North York, ON, Canada; 3 The Department of Political Studies, 6515 Trent University, Peterborough, ON, Canada

**Keywords:** Afghan refugees, resettlement, mental health, health care access, integration, settlement

## Abstract

**Background:**

Decades of war and political instability have forced millions of Afghans to flee from their homes, resulting in one of the world's largest humanitarian crises. Many refugees have resettled in North America, particularly in Canada and the United States, where they have encountered numerous psychosocial and systemic barriers to adapting to their new environment.

**Objective:**

This scoping review aims to explore the settlement experiences of Afghan refugees in North America, synthesize existing evidence on integration challenges, and identify key gaps in the literature.

**Methods:**

Following Arksey and O’Malley's methodological framework, six electronic databases were searched for relevant literature published between 2014 and 2024, which focused on Afghan refugee settlement experiences in the North American context. Seventeen eligible studies were included in the final review.

**Results:**

Mental health emerged as the most studied topic, with Afghan refugees experiencing moderate to high rates of psychological distress, depression, and post-traumatic stress disorder. Key risk factors included female gender, older age, pre-migration trauma, financial constraints, and social isolation. Protective factors, such as, strong social support networks, English language proficiency, and gainful employment were associated with improved mental health outcomes. In spite of the generally positive healthcare experiences, Afghan refugees encountered language barriers, limited health literacy, transportation difficulties, and cultural misunderstandings with healthcare providers.

**Conclusion:**

Afghan refugees in North America face complex and intersecting barriers to health and healthcare access, and integration. Current literature by and large focuses on mental health, and there is an urgent need to expand research in other important areas of post-migration and (re)settlement.

## Background

Afghanistan is a landlocked country in south-central Asia with a diverse population of approximately 43 million people (Worldometer, 2025). Afghanistan has endured over four decades of conflict, political instability, natural disasters, and economic hardship. The successive cycles of violence and instability, such as the Soviet invasion in 1979, the Afghan civil wars of the 1990s, and the rise of the Taliban regime, have contributed to the tremendous political, economic, and social instability that this country has faced for decades. This situation is exacerbated by other complex policy decisions such as the withdrawal of U.S. forces from Afghanistan in 2021, the reduction in international financial aid, and the Taliban's most recent resurgence to power. These events have created one of the most devastating and most enduring displacement crises globally (Center for Preventive Action, 2024; United Nations High Commission for Refugees (UNHCR, 2024a)).

A refugee is defined as a person forced to flee their home country due to persecution or fear of persecution, war, and violence based on membership in a particular social group (UNHCR, n.d.). Currently, an estimated 10.9 million Afghans have been forcibly displaced within their country and across international borders. The UNHCR's (2023) Global Trends report revealed that more than 6.4 million Afghan refugees have fled to other countries, making Afghans the third-largest displaced population in the world (UNHCR, 2024a). The Taliban's return to power in 2021, following the withdrawal of U.S. and allied forces, has only further intensified this displacement crisis and economic collapse. The regime's strict conservative Islamic ideologies have imposed significant restrictions on fundamental human rights and freedoms, access to education, employment opportunities, public spaces for women and girls, and marginalization of ethnic minorities (Amnesty International, 2023; Center for Preventive Action, 2024). Women and children have been disproportionately affected during the Taliban's resurgence, and they represent up to 80% of Afghans who have been forcibly displaced since May 2021 (UNHCR, 2021). Canada is a global leader in refugee resettlement and has consistently received the most or second-most (re)settled refugees since 2016, accounting for 25% to 42% of all globally (re)settled refugees annually (Government of Canada, 2024a). Canada has resettled 55,195 Afghan refugees since 2021, exceeding its initial commitment of 40,000 Afghan refugees. Most Afghan refugees have (re)settled in the provinces of Ontario, British Columbia, and Alberta. The Greater Toronto Area accounts for more than 50% of Canada's total Afghan refugee population (Government of Canada, 2024b).

After arriving in the host countries, refugees often encounter several significant psychosocial barriers to resettlement, including language, cultural, housing, transportation, employment, and economic challenges (Henry et al., 2019). Resettled refugees experience worse physical health outcomes, a higher prevalence of chronic diseases, and an increased risk of developing conditions such as hypertension and diabetes as compared with the non-refugee immigrant and locally-born populations residing in host countries (Kumar et al., 2021). Among refugee groups, Kohlenberger et al. (2019) found that Afghan refugees perceived their health status to be low. The authors suggested that this may be due to the prolonged, harsh migration experiences, pre-migration trauma, social exclusion, and challenges with acquiring legal status, all of which are more common among Afghan refugees. These challenges are further compounded by difficulties navigating the healthcare system and obtaining care, limited access to interpretation services, lack of culturally safe care, experiences of discrimination and racism, and long wait times for healthcare services (Coumans & Wark, 2024; Kohlenberger et al., 2019). The cumulative effects of these healthcare system barriers hinder refugees’ ability to seek, utilize, and benefit from essential healthcare services, which ultimately impedes their overall health and well-being, and successful integration into the host society.

Forced displacement has profound and lasting impact on the mental health of individuals and families who experience forced migration. Refugees who have resettled in high-income countries are at substantially increased risk of developing anxiety, depression, and post-traumatic stress disorder (PTSD) in comparison to individuals born in the host countries (Henkelmann et al., 2020). Particularly, decades of conflicts and political upheavals have exposed Afghans to numerous extreme traumatic events such as persecution, kidnapping, and separation from family members. Such exposures create substantial vulnerability to a wide range of mental health conditions (Mesa-Vieira et al., 2022; Miller et al., 2018).

The scale of traumatic exposure among Afghans has been well documented. In a large national study that surveyed 4445 participants across eight regions in Afghanistan, 86.16% of the sample reported having personally experienced or witnessed at least one traumatic event in their lifetime. Of this, 64.67% reported personally experiencing trauma, 78.48% witnessed trauma, and 60.77% experienced collective violence related to armed conflicts (Kovess-Masfety et al., 2021). A recent systematic review further reported that up to 1 in 3 refugees experience depression and/or PTSD, while 1-2 in 10 suffer from anxiety disorders (Henkelmann et al., 2020).

Similar to other displaced populations, Afghan refugees exhibit moderate to high prevalence of depressive and post-traumatic symptomatology levels (Alemi et al., 2014). Prevalence estimates of anxiety, depression, and PTSD among Afghan refugees vary widely, with reported rates ranging from 42% to 71.94% for anxiety (Kurt et al., 2022; Neyazi et al., 2024), 50.3% to 72.05% for depression (Kurt et al., 2022; Neyazi et al., 2024), and 31.1% to 48.8% for PTSD (Andisha & Lueger-Schuster, 2024; Hamrah et al., 2021). Female Afghan refugees are especially vulnerable as they are twice as likely to have anxiety, depression, and PTSD in comparison to their male counterparts (Kurt et al., 2022). More broadly, refugee women tend to experience higher prevalence and greater severity of mental health conditions compared to refugee men (Kovess-Masfety et al., 2021; Vallejo-Martín et al., 2021).

Given the ongoing global refugee crisis, there is an urgent need to strengthen understanding of the settlement experiences of Afghan refugees, a vulnerable population that continues to face prolonged displacement and complex resettlement challenges. Although several studies have examined multiple aspects of refugee integration, the literature specific to Afghan refugees in North America remains fragmented, dispersed across disciplines, and highly variable in scope. Existing research covers a wide range of domains including mental health, food security, acculturation, social integration, and family dynamics, yet no comprehensive synthesis has examined how these experiences are represented collectively in the literature.

A scoping review approach was therefore selected to address these gaps. This methodology is particularly appropriate given that the existing literature on Afghan refugee resettlement is heterogeneous, emergent, and methodologically diverse, making it unsuitable for a systematic review at this stage. Scoping reviews enable researchers to map the breadth and nature of available evidence, clarify key concepts, identify gaps in knowledge, and synthesize findings across a wide range of study designs (Gottlieb et al., 2021). Guided by Arksey and O’Malley's (2005) framework, the present review aims to (1) explore and expand understanding of Afghan refugee settlement experiences in North America; (2) examine the facilitators, barriers, and challenges experienced by Afghan refugees during their resettlement process; and (3) identify gaps in current literature. Using the Population-Concept-Context (PCC) framework, this review focuses on Afghan refugees and asylum seekers (population); their resettlement experiences (concept); within the North American context, specifically Canada, United States, and Mexico (context).

## Materials and Methods

This study utilized the methodological framework outlined by Arksey and O’Malley (2005) for conducting scoping reviews and reported in accordance with the PRISMA-ScR (Preferred Reporting Items for Systematic reviews and Meta-Analyses extension for Scoping Reviews) guidelines. The framework consists of five stages: identifying the research question; conducting a comprehensive search for relevant studies; selecting appropriate studies based on predefined criteria; charting the data; and collating, summarizing, and reporting the results. This method was chosen for its suitability in capturing a wide range of research and synthesizing findings to identify knowledge gaps and inform future studies. A review protocol was not developed for this scoping review. No registration information is available.

### Search Strategy

The following electronic databases were searched for relevant articles: CINAHL, EBSCOHost, EMBASE, Medline, ProQuest and PubMed. A comprehensive list of search terms was developed based on the key concepts of the research question. Thesauri were used to retrieve synonyms and related terms. Medical Subject Headings (MeSH) were used when available. The following key terms were utilized to conduct the search: “Afghan”, “refugee”, “immigrant”, “migrant”, “emigrant”, “settlement”, “resettlement”, “experiences”, “challenges”, “barriers”, and “difficulties”. Search terms were combined using Boolean operators and wildcards (*) were used to account for different spellings and pluralizations. Table 1 details the search strategy used.

**Table 1.** Search Strategy of Each Database.

Database	Search Strategy	Results
CINAHL	(MH “Refugees”) AND (MH “Afghan persons”) AND (experiences OR challenges OR barriers OR difficulties)	0
	(MH “Refugees”) AND (MH “Afghan persons”)	25
EBSCOhost	(Afghans OR Afghan) AND (migration OR immigration OR emigration OR refugee) AND resettlement refugee)	16
EMBASE	Afghan* AND (refugee OR immigra* OR migra* OR emigra* OR settlement OR resettlement) AND (experiences OR challenges OR barriers OR difficulties)	126
Medline	Afghan* AND (refugee OR immigra* OR migra* OR emigra* OR settlement OR resettlement) AND (experiences OR challenges OR barriers OR difficulties)	173
Proquest	Afghan AND refugees AND (experiences OR challenges OR barriers OR difficulties)	576
Pubmed	Afghan* AND (refugee OR immigra* OR migra* OR emigra* OR settlement OR resettlement) AND (experiences OR challenges OR barriers OR difficulties)	326
	Total	1217

**Table 2.** Summary of Selected Articles.

Authors and Year	Country	Topic	Aim	Method	Population and Sample Size	Results
Soltan et al. (2023)	Canada	Acculturation	Examine impact of acculturation gaps between Afghan refugee young adults and their parents on family relationships, adaptation outcomes, and subjective wellbeing in Canada.	Qualitative	148 participants: 77 emerging adults, 68 parents, and 3 caregivers	Emerging adults in Afghan refugee families who maintained proficiency in Farsi and a strong Afghan cultural identity experienced better mental health and family relationships. Parents’ acculturation to Canadian culture significantly supported their children's psychological adaptation. A large gap in Canadian identity between parents and emerging adults led to poorer family cohesion and increased intergenerational conflict.
Goliaei et al. (2023)	U.S.	Food Security	Investigate Afghan refugees’ food access and insecurity in the San Joaquin Valley, California.	Qualitative	12 key informants and 12 Afghan refugee families	Afghan refugee newcomers faced significant challenges accessing food due to unfamiliarity with grocery store systems, limited transportation, high costs, and a lack of culturally or religiously appropriate options (halal food). Many avoided food banks because the items provided did not meet their dietary restrictions. They expressed a need for better financial literacy support and access to affordable, culturally suitable groceries.
Abuali et al. (2024)	U.S.	Health Profile	Understand the physical and psychosocial health needs of newly arrived Afghan refugee children.	Retrospective study	121 recent Afghan refugee children	A review of 121 patient charts revealed high rates of health issues among Afghan refugee children, including malnutrition (25%); dental caries (74%); treatable infectious diseases such as schistosomiasis, strongyloidiasis, tuberculosis, and leishmaniasis; and mental health symptoms. Most parents and children did not speak English, and barriers to healthcare access included limited English proficiency and lower parental education levels.
Siddiq et al. (2022)	U.S.	Healthcare Access and Experiences	Explore sociocultural factors that influence mammography and colonoscopy screening behaviors among Afghan refugee women.	Focused ethnography design	19 Afghan women over the age of 50	Afghan refugee women often perceived cancer as a fatal disease, leading to fear, shame, and avoidance of preevntive screening. This was influenced by negative healthcare experiences, such as miscommunication and language barriers. However, support from family, reminders from providers, care by female practitioners, and spiritual coping strategies like duaa fostered resilience and encouraged engagement in cancer screening.
Siddiq et al. (2023)	U.S.	Healthcare Access and Experiences	Explore older Afghan refugee women's perceptions of individual and sociocultural factors of health and health care experiences.	Focused ethnography design	14 Afghan women over 50, 5 family members, 8 community informants	Older Afghan refugee women's health experiences were shaped by five themes: health promotion thrrough religion, central role ofd family, ongoing displacement stressors, challenges navigating the health care system, and miscommunication from healthcare providers.
Worabo et al. (2024)	U.S.	Healthcare Access and Experiences	Investigate Afghan women's maternal health experiences in South Texas.	Qualitative descriptive design	20 Afghan women who gave birth in the US within the past 2 years	Afghan refugee women in the U.S. reported largely positive maternal healthcare experiences, which contrasts the challenges they experienced in Afghanistan. They shared preference for female provideres, need for contraceptive education, and difficulties navigating the healthcare system. Some shared concerns about poor birth outcomes, language barriers, and structural barriers (e.g., transportation, finances).
Rosenberg et al. (2022)	U.S.	Healthcare Access and Experiences, Mental Health	Identify potential interventions to increase access to needed care and services for Afghan refugee children.	Community-based participatory research, grounded theory	10 recently-arrived Afghan refugee families (9 mothers and 10 fathers)	Four themes: a shift in parental focus toward children's development as safety improved; acculturation stress related to technology use and preserving traditions; gradual adjustment to the U.S. support system amidst feelings of isolation; and growing trust in the more involved and coordinated U.S. healthcare system.
Rosenberg et al. (2024)	U.S.	Healthcare Access and Experiences, Mental Health	Analyze the perspectives and experiences of Afghan families and refugee-serving stakeholders regarding mental health program. To determine factors of successful implementation of preventive mental health intervention.	Qualitative thematic analysis	6 refugee-serving stakeholders and five refugee parents.	Five key themes were identified in supporting refugee families: cultural humility and mutual learning in discussing emotions; adequate support networks; foster multidirectional communication among children, families, and educators; differing perspectives between families and stakeholders on intervention outcomes; and timely, culturally-informed interventions.
Quirke (2014)	Canada	Information practices	Explore the information practices of Afghan newcomer youth in Canada, focusing on two contexts: leisure activities and settlement.	Qualitative Exploratory	7 Afghan newcomer youth in Canada (ages 12 to 24)	Afghan newcomer youth in Canada experienced significant challenges in accessing information due to language barriers, unfamiliar systems, and social isolation, often relying on informal networks and technology. Their leisure activities were shaped by cultural tensions and adaptation struggles, highlighting the need for more culturally responsive and accessible information services.
Alemi et al. (2015)	U.S.	Mental health	Investigate psychological distress symptoms and examine variables that correlate with and predict psychological distress levels among Afghan refugees.	Cross-sectional	130 Afghan refugees (74 males and 56 females)	Low levels of distress observed in this sample, possibly reflecting improved safety. Symptoms like insomnia, headaches, and irritability were common. Factors that predicted distress: female, widowed, financial difficulties, disabled, and low education attainment. Factors that did not predict distress: acculturation, perceived social support
Alemi and Stempel (2018)	U.S.	Mental health	Investigate effects of perceived discrimination, distress, and post-settlement factors on the mental health of Afghan refugees.	Cross-sectional	250 Afghan resettled refugee adults	Higher psychological distress was significantly related to perceived discrimination, pre-resettlement trauma, and strong intra-ethnic identity. Social support and ethnic integration reduced distress.
Stempel et al. (2016)	U.S.	Mental health	Examine the moderating/mediating effects of gender on distress and resilience among Afghan refugees.	Cross-sectional	259 Afghan refugee adults	Higher distress levels were significantly associated with perceived discrimination, pre-resettlement trauma, and dissonant acculturation. English proficiency and family ties offer protective effects, especially for women. Gender ifferences revealed that English ability reduced distress more for women, while traditional gender ideologies and extended family ties increased distress for men.
Ahmad et al. (2020)	Canada	Mental health	Explore effects of social support, coping on PTSD symptoms among Afghan refugees in Canada.	Cross-sectional	49 Afghan refugees adults resettled in Canada	PTSD prevalence in this sample was 53.1%, higher among those who were older, unemployed, and self-reported poor/fair health. Increased perceived social support significantly buffered PTSD symptoms and was associated with better coping and self-rated health.
Kirsch et al. (2024)	U.S.	Mental Health	Explore the relationship between collective trauma and mental health outcomes within Afghan refugee population.	Mixed method: Community-based participatory research, cross-sectional	173 recently resettled Afghan refugees	Trauma and mental health outcomes varied by visa status, gender, displacement, English proficiency, and income level. Humanitarian parolees, women, low-income individuals, and minority ethnic groups experienced higher levels of mental health symptoms and traumas.
Alemi et al. (2017)	U.S.	Mental health	Investigate beliefs about depression among Afghans residing in the San Diego area.	Qualitative	93 Afghan refugees (50 men and 43 women)	Both genders shared core beliefs about causes, risk factors, and treatments for depression. Women identified more causes and symptoms and favored religious or herbal remedies, while men linked depression to cultural elements like Afghan music and their gender. Women showed greater cultural knowledge and reported higher psychological distress related to socioeconomic and acculturation challenges.
Alemi et al. (2016)	U.S.	Mental health	Examine how Afghan refugees conceptualize depression and describe their experiences with depressive symptoms.	Qualitative descriptive approach	18 Afghan refugee adults (11 males, 7 females)	Afghan refugees reported pre-migration traumas such as war, family separation, and difficult journeys, compounded by post-resettlement stressors including cultural adjustment, financial hardship, housing issues, and loss of Afghan identity and values. Depression was described using culturally specific terms and linked to both physical and psychological consequences. Coping strategies emphasized family unity, social connection, prayer, and finding purpose through their children's successes.
Nourpanah (2014)	Canada	Social Integration	Analyze how government-assisted Afghan refugees adapt to life in Canada.	Qualitative	10 Afghan government-assisted refugees	Afghan families in Canada maintained strong visual and cultural ties to their heritage while also adapting to new social norms. Mutual accommodation and negotiation between parents and children on issues such as religion, gender roles, and education. Expression of religiosity varied as some families found comfort and identity in faith, while others distanced themselves from religion, viewing it as a symbol of past constraints.

### Inclusion and exclusion criteria

The inclusion criteria for this study were carefully defined to ensure relevance and applicability. Studies written in English, focusing on Afghan refugees, conducted in the North American context (Canada, United States, and Mexico), and published between 2014 and 2024 were eligible. Acceptable study designs included quantitative, qualitative, mixed methods, systematic reviews, scoping reviews, meta-analyses, dissertations, and grey literature. Articles published prior to 2014 and those written in languages other than English were also excluded.

### Selection Process and Data Extraction

**Figure 1.**
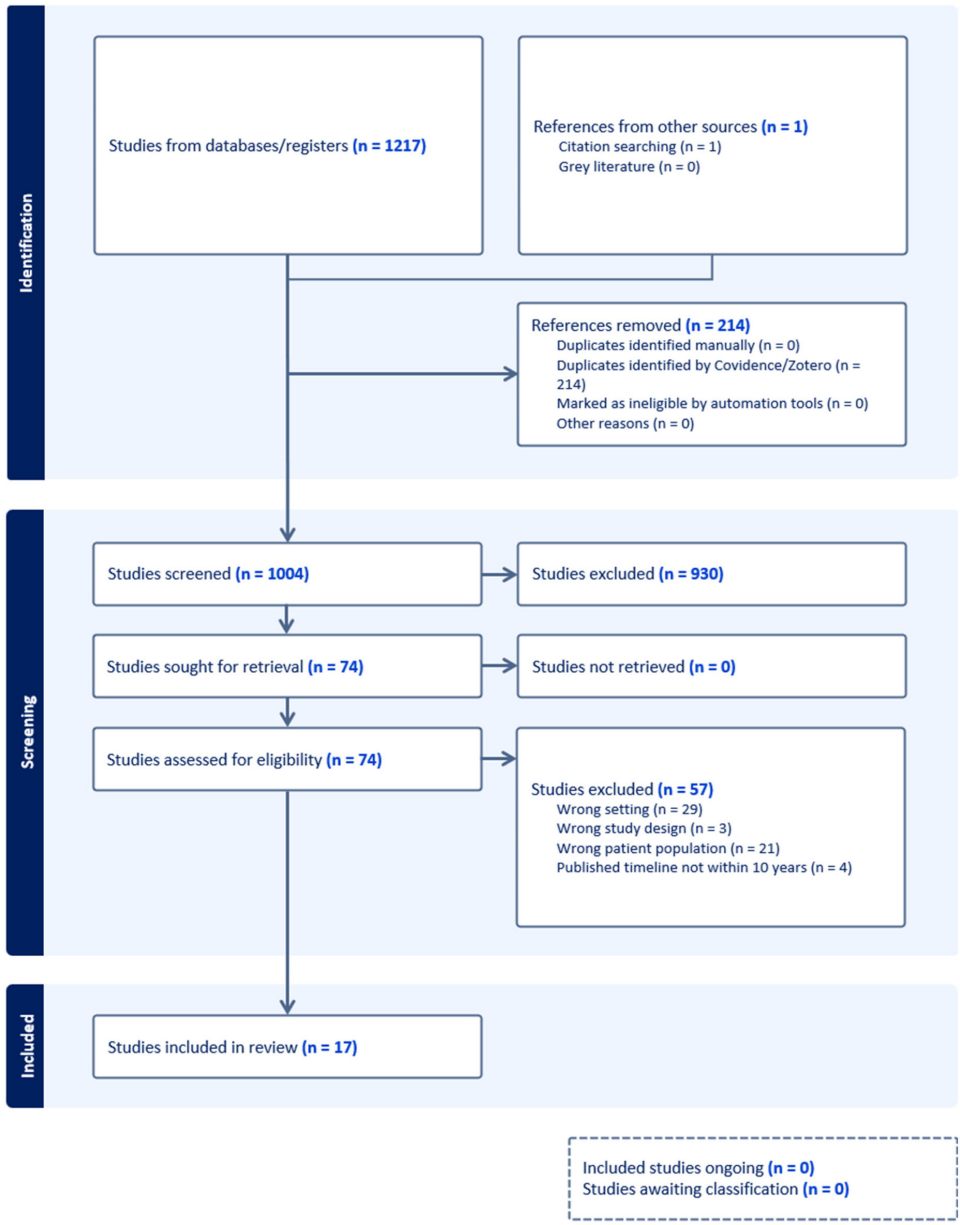
PRISMA diagram.

A total of 1,217 references were imported to Covidence for screening, and 114 references were removed as duplicates. The resulting 1,003 articles were subjected to title and abstract screening for relevance by two independent reviewers (BW and PJ). During the initial screening, 930 studies were excluded as they did not meet the inclusion criteria. Level 1 screening of Titles and Abstracts sought articles that were: …Level 2 screening of full text sought articles that were … Seventy studies were subjected to full-text review by two independent reviewers. Disagreements were resolved by discussions among the research team. Seventeen articles were included in the final review. Consistent with the Arksey and O’Malley scoping review methodology, no formal appraisal of study quality was conducted to capture a broad range of available evidence. The selection process is captured in the PRISMA diagram as seen in Figure 1.

Data charting and extraction was independently conducted by two reviewers (BW and PJ) using a table developed and reviewed by the research team. The table captured details of each study, including author(s), year, country, setting/discipline, purpose, study design, population and sample size, data collection and analysis methods, results, and conclusions.

### Data Analysis

All included studies were charted and analyzed using an inductive thematic approach., Using the data that had been charted, two independent reviewers (BW and PJ) conducted inductive coding of the extracted data in order to develop themes. Each reviewer coded the findings separately, then compared and reconciled discrepancies through regular discussions. The finalized codes were grouped into overarching themes that captured key aspects of Afghan refugees’ post-settlement experiences.

## Results

Across the final 17 included studies, Afghan refugees’ settlement experiences were characterized by intersecting challenges related to mental health (*n* = 7), healthcare access (*n* = 5), health status (*n* = 2), food security (*n* = 1), social integration (*n* = 1), and information practices (*n* = 1) (Table 2).

### Mental Health

Mental health emerged as the most extensively examined and central aspect of the settlement experience for Afghan refugees. The literature consistently highlighted a range of challenges and contextual factors that shaped psychological well-being and influenced adaptation within host communities. These findings are summarized below.

#### Psychological Distress

There is conflicting data on the levels of distress experienced by resettled Afghan refugees. Alemi et al. (2015) reported an overall low level of distress among 130 Afghans in San Diego County, U.S. Meanwhile, a sample of 259 Afghan refugees in Northern California, U.S., reported low to moderate levels of distress (Stempel et al., 2016).

Factors associated with lower distress levels include lower age, younger age at migration, higher educational attainment, English language proficiency, gainful employment, increased perceived social support levels, and Pashtun ethnicity (Alemi et al., 2015; Alemi & Stempel, 2018; Kirsch et al., 2024; Stempel et al., 2016). Variables associated with higher distress levels are women, older age, unemployment, widowed, pre-settlement stressors, residence on a military base, low income or financial difficulties, perceived discrimination, dissonant acculturation, elevated perceived stress, and ethnic minorities (e.g., Tajik, Hazara, Nuristani, Uzbek) (Alemi et al., 2015; Alemi & Stempel, 2018; Kirsch et al., 2024; Stempel et al., 2016).

Several studies have found that women consistently reported higher distress levels and increased frequency of distress symptoms compared to men (Alemi et al., 2015; Alemi et al., 2014; Stempel et al., 2016). Stempel et al. (2016) further investigated the moderating effects of gender on several variables. The authors found that increased distress levels were observed among men with traditional gender ideologies and women with egalitarian views. Strengthening family connections substantially reduced stress levels in women, whereas for men, an increase in family ties slightly raised distress levels. Maintaining extended family ties was the most stressful for men (Stempel et al., 2016). English proficiency notably lowered distress levels for women and only marginally decreased distress for men. Regardless, promoting language acquisition is of utmost importance as English abilities enhance employment prospects and income, which are significant predictors of distress (Stempel et al., 2016). In a separate study, English language proficiency was strongly associated with lower distress symptoms, improved mental health outcomes, and fewer post-migration difficulties for both genders (Kirsch et al., 2024). Dissonant acculturation only increased men's distress levels and not women's (Stempel et al., 2016).

According to Alemi et al. (2015), three demographic variables (e.g., female, widowed, and unable to pay monthly bills) were the most substantial contributors to the overall model of predicting psychological distress. Furthermore, acculturation and perceived social support did not predict psychological distress (Alemi et al., 2015). Older Afghan women reported significant concerns over their mental health and ongoing stress. Their high levels of distress were attributed to pre-migration trauma (e.g., war and family separation) and post-migration stressors (e.g., shifting gender roles, cultural conflicts, family conflicts, limited language proficiency, and social isolation) (Siddiq et al., 2023). In an American qualitative study, parents identified their children's increased screen time and difficulties maintaining religious practices as acculturation stressors (Rosenberg et al., 2022). Equally important, perceived discrimination and pre-migration stressors are also significant predictors of psychological distress (Stempel et al., 2016). This finding is corroborated by a study conducted by Alemi and Stempel (2018) in which perceived discrimination is significantly associated with elevated distress levels (Alemi & Stempel, 2018). The authors proposed that pre-settlement trauma exacerbates the detrimental effects of perceived discrimination, leading to elevated distress levels among Afghan refugees. Individuals with strong ethnic identities are also more prone to the adverse effects of discrimination, whereas those with ethnic integration orientation have reduced distress levels (Alemi & Stempel, 2018).

#### Depression

Afghan refugees conceptualized that depression is caused by pre-migration traumas (e.g., war, family separation, home invasions, interrogation) and post-resettlement stressors (e.g., status loss, unemployment, financial constraints, cultural conflicts, cultural identity loss, linguistic challenges, transportation, social isolation, intergenerational issues, and uncertainties about the future) (Alemi et al., 2016, 2017). According to Alemi et al. (2016), pre-migration trauma is a common experience shared by all participants regardless of socioeconomic background, and many described family separation as the most detrimental source of trauma. Risk factors for depression include identifying as female, having a chronic comorbidity, living with a depressed family member, and older age (Alemi et al., 2017). In a sample of 93 respondents, Alemi et al. (2017) found that female participants were more likely to have depression and reported twice as many depressive symptoms compared to men. Despite these gender differences, Alemi et al. (2017) concluded that both Afghan men and women shared a common set of core beliefs about depression. Both genders attributed depression to pre-migration traumatic experiences and post-migration stressors and identified similar treatment options. Interestingly, both genders in this study did not view depression as hereditary, which contrasts with a modern Western view where genetic predisposition is a significant contributing factor. Respondents shared culture-specific expressions to describe depression, which include *asabi* (irritability), *gham* (unexplained sadness), and *goshagiry* (self-isolation) (Alemi et al., 2016). Afghan men and women perceived depression as a treatable condition that will not resolve without interventions. Treatment options described by Afghan refugees are psychiatric consultation, antidepressants, relaxation, religious activities, family connection and community engagement, and treatment of underlying illnesses (Alemi et al., 2016, 2017).

#### Post-Traumatic Stress Disorder

Similar to estimates in other refugee populations, PTSD is highly prevalent in Afghan populations. In a survey of 49 Afghan refugees in Canada, 53.1% of respondents had scores deemed detectable for PTSD (Ahmad et al., 2020). Risk factors for PTSD in Afghan refugees include older age, unemployment, and self-rated poor or fair health. Individuals older than 45 years of age had a PTSD prevalence of 80% compared to 36.8% in those 30 years or younger (Ahmad et al., 2020). Variables that were significantly associated with lower PTSD prevalence are employment and perceived social support. Social support significantly reduced PTSD severity across all domains of re-experiencing, avoidance, and arousal. Coping is mediated through social support, and having children is related to higher coping scores. English language proficiency is a strong predictor of social support (Ahmad et al., 2020). This again highlights the critical role of language acquisition in mitigating the risks of developing mental health symptoms and conditions. In an American study by Kirsch et al. (2024), visa status emerged as a crucial factor in relation to trauma symptoms. Specifically, individuals with Asylee status and humanitarian parolees reported higher levels of individual and collective trauma symptoms, worse mental health outcomes, and increased post-migration challenges (Kirsch et al., 2024).

### Health Status, Healthcare Practices, and Healthcare Experiences

Six studies investigated healthcare experiences and psychosocial factors or barriers to healthcare access among Afghan women (*n* = 3) or children (*n* = 3) in North America (Abuali et al., 2024; Rosenberg et al., 2022; Rosenberg et al., 2024; Siddiq et al., 2020; 2023; Worabo et al., 2024). There were no studies included in this review that explored the healthcare experiences of men. The most frequently cited barriers to healthcare access were language, miscommunications, lack of health literacy, lower levels of education, transportation, and financial constraints (Abuali et al., 2024; Siddiq et al., 2020). Women consistently express a preference for female providers and interpreters when seeking healthcare (Siddiq et al., 2020; Worabo et al., 2024). Worabo et al. (2024) found that Afghan women who accessed maternal services in the United States generally reported positive and pleasant experiences. They expressed appreciation for high-quality care, attentive providers, and the wide availability of resources that supported their prenatal, birthing, and postpartum needs. They highlighted contrasts with Afghanistan, where economic hardship, limited access to prenatal care, and heavy physical work were common challenges. While the majority described their experiences as overwhelmingly positive, some reported discomfort with male healthcare providers and a need for better contraceptive education. Women cited language barriers as the most challenging issue. While interpretation services were often available, some women expressed discomfort with male interpreters when discussing sensitive reproductive health topics. Women also highlighted the importance of respecting their cultural preferences. Lack of transportation and financial constraints were identified as structural barriers. Several women noted their inability to operate vehicles and reliance on their husbands for transportation (Worabo et al., 2024). Afghan women underutilize preventive cancer screening, as many women reported limited knowledge about cancer and screening recommendations. Some women perceived cancer as a “dangerous and deadly disease” and viewed screening as a confirmatory diagnosis (Siddiq et al., 2022, p. 355). They often avoided discussions of cancer due to fear and shame and often kept them private to minimize worries among close family. When accessing screening services, women experienced language barriers, miscommunications, communication delays, and unclear explanations of the screening process or results. These negative healthcare experiences led to frustration and further deterred women from accessing preventive services. As one participant stated, “it's not the religion - it's the language”, highlighting the importance of language in shaping healthcare behaviours and access (Siddiq et al., 2022, p. 359). Women also described sources of empowerment that encouraged them to navigate the stressful screening process. These factors are family support, reminders from healthcare providers, and prayers (*duaas*) (Siddiq et al., 2022).

Older Afghan women recognized the importance of family involvement and religion in maintaining their health and health practices. They viewed health holistically and identified family as a motivating factor in promoting their health. Over half reported at least one chronic health condition, including diabetes, cancer, hypertension, arthritis, chronic pain, or mobility challenges. Mental health was cited as a significant concern, compounded by pre-migration trauma, resettlement hardships, cultural challenges, family conflicts, and social isolation. Older Afghan women highlighted the critical role of family members in their healthcare experiences, often relying on relatives for decision-making, language interpretation, transportation, advocacy, and support. While community resources were available in Afghan communities, not all women had access to these supports, and frailty or chronic health conditions often limited their participation in community activities. Miscommunication with healthcare providers and unmet treatment expectations led to distrust and dissatisfaction among older Afghan women (Siddiq et al., 2023). Two studies examined families’ perspectives on the access and implementation of pediatric mental health services. Similar to Afghan women who utilized maternal healthcare services, parents shared appreciation for consistent, coordinated visits and reported trust in the U.S. healthcare system. The same parents also described a gradual transition from initial social isolation to building a growing, robust local support system (Rosenberg et al., 2022). The same authors further explored determinants of successful preventive mental health interventions. The main recommendations were to promote the normalization of discussing emotions through cultural humility, bridge communication gaps between children and parents, maintain support networks to minimize miscommunications, and ensure timely cultural adaptation of interventions (Rosenberg et al., 2024).

### Other Post-Migration Challenges

In the post-migration setting, researchers have investigated other pertinent areas such as food insecurity, integration and acculturation, leisure activities, and information practices using qualitative research methods. For example, Goliaei et al. (2023) conducted a qualitative study on the experiences accessing food resources among recently (re)settled Afghan refugees accessing food resources in the United States. The study emphasized several individual, environmental, and systemic barriers that refugees faced, including difficulties navigating grocery stores, lack of transportation, language barriers, high costs, limited availability of culture-specific foods (e.g., Halal), and low financial literacy to manage monthly budgets. Additionally, Afghan refugees were found to underutilize food support programs, often due to unfamiliarity with local selection, scarcity of culturally appropriate options, and discomfort participating in these programs. Families reported a preference for shopping at ethnic stores, despite the higher pricing and lower availability of items (Goliaei et al., 2023).

Similar to other age groups, Afghan newcomer youths faced post-settlement stressors, including language barriers, cultural conflicts, legal status challenges, social isolation, family separation, and being minors with adult responsibilities. Afghan youths relied heavily on family, friends, settlement workers, and social media to exchange information. Unaccompanied youths often maintained secrecy and were reluctant to discuss information with families back home. The same youths engaged in leisure activities such as being with friends and family, participating in religious activities, *mehmani* (Afghan visiting ritual), writing poetry, attending school, and playing sports. These information practices and leisure activities shape how youths maintain their distinct Afghan cultural identity while integrating a Canadian identity (Quirke, 2014).

As Afghan refugees resettle in host societies, distinct integration practices emerged in North American societies, highlighting the intricate relationship between cultural identity and adaptation. Many families maintained solid connections to their Afghan heritage and identity through language, traditional attire and decorations, and participation in religious festivities. Similarly, many changes were also evident as gender roles, traditional norms, and educational priorities began to shift. For example, families permitted children and young adults to independently decide on their religion, marriage, education, and careers. While parents reported accepting their children's choices, this did not prevent them from imparting their values and traditions. Patriarchal norms are increasingly challenged as families prioritize higher education for girls and move away from traditional gender expectations. Most refugees described religion as a source of strength, comfort, and familiarity. Some have denounced this practice as they perceived religion as constrictive, obsolete, and a cause of suffering in Afghanistan (Nourpanah, 2014). As younger generations readily adapted to the dominant Western culture, this often created generational differences that disrupted family cohesiveness and promoted feelings of estrangement (Alemi et al., 2016; Soltan et al., 2023). Young adults with stronger Farsi fluency and a deeper understanding of Afghan culture reported more positive family relationships and increased subjective well-being. Parents more acculturated to Canadian culture were better able to guide their children through the adaptation process, thereby improving their children's subjective well-being (Soltan et al., 2023). Overall, Afghan refugees have demonstrated significant resilience in overcoming the challenges of resettlement and adapting to host societies.

## Discussion

This scoping review aimed to examine the settlement experiences of Afghan refugees in the North American context. The included studies highlighted several issues and challenges that Afghan refugees have encountered in navigating new sociocultural, economic, and healthcare landscapes. The current review reinforced the existing evidence on the multifaceted nature of refugee resettlement, emphasizing the intersectionality of biopsychosocial and broader systemic factors in shaping the integration process. Specifically, this review has identified key challenges to mental health, health status, and healthcare experiences.

As expected, the Afghan refugee population resettled in Canada and the U.S. experienced elevated rates of mental health conditions, including psychological distress and PTSD (Ahmad et al., 2020; Kirsch et al., 2024). The prevalence of PTSD in this review is 53.1%, which is significantly higher than the overall PTSD prevalence of 7.7% among Canadian adults (Government of Canada, 2024c). While the prevalence of PTSD appears notably greater in the current review, this should be carefully interpreted as this estimate is based on a small, non-randomized sample of 49 adult participants at a community health clinic. This trend aligns with global data from countries such as Pakistan, Turkey, Australia, Germany, Austria, and other high-income countries (Habib & Zulfiqar, 2025; Hamrah et al., 2021; Kurt et al., 2022; Walther et al., 2020). However, prevalence statistics in research studies differ across samples, geographical locations, and study methodologies.

It is unsurprising that PTSD is one of the most prevalent mental health conditions among resettled Afghan refugees. Four decades of war and political upheaval and socioeconomic strife have repeatedly exposed Afghan communities to numerous traumas, including organized violence, poverty, economic instability, lack of basic needs, forced displacement, poverty, and loss of housing (Alemi et al., 2023; Kovess-Masfety et al., 2021). Kurt et al. (2022) found that in a sample of 785 Afghans, 84.6% of the participants reported experiencing at least two traumatic events either in their home country or during their journey to Turkey. This already vulnerable and highly traumatized group is further subjected to additional stressors in the post-migration setting. After resettlement, refugees often experience discrimination, language barriers, unemployment, financial difficulties, social isolation, housing, cultural adjustment, and legal status (Ahmad et al., 2025; Gleeson et al., 2020). Research has consistently demonstrated that repeated exposures to pre-displacement trauma and post-settlement stressors are the most significant predictors of mental health conditions among refugee groups (Andisha & Lueger-Schuster, 2024; Hynie, 2018; Knipscheer et al., 2015). Some authors have further elucidated that post-migration stressors have a larger impact on refugees’ mental health and play a critical role in mediating the effects of pre-migration trauma (Chen et al., 2017; Andisha & Lueger-Schuster, 2024). The current review supports this, as pre-migration trauma and post-migration stressors were frequently cited as the main risk factors that contribute to adverse mental health outcomes (Alemi et al., 2016, 2017; Alemi & Stempel, 2018; Stempel et al., 2016). Equally important, PTSD often signifies the conclusion of a traumatic event, it is crucial to acknowledge the ongoing, lived experience of trauma among Afghan refugees. Concepts like continuous trauma, collective trauma, and intergenerational trauma better reflect the persistent reality of violence, poverty, and instability that Afghans face daily (Ullah et al., 2023). Continuous trauma, in particular, describes trauma as ongoing, unpredictable, and occurs simultaneously with the present and future (Eagle & Kaminer, 2013). Given the interconnectedness of pre- and post-migration stressors, host countries must mitigate adverse mental health outcomes by implementing holistic, culturally relevant psychosocial interventions that address the cumulative effects of both. An effective approach must acknowledge the enduring impact of pre-migration traumas while simultaneously reducing the socioeconomic and structural stressors encountered in the post-migration context.

Despite high rates of PTSD, the current review found conflicting data on psychological distress levels among resettled Afghan refugees. This inconsistency may be attributed to a small sample size, non-randomized sampling, the sociopolitical context of the host communities, the availability of community resources, under-recognition or underreporting of symptoms, and the use of instruments that have not been validated for Afghan populations. Specifically, Stempel et al. (2016) measured distress using the Talbieh Distress Inventory, a 24-item self-report questionnaire designed broadly for immigrants and validated in a sample of Israeli immigrants to Russia (Ritsner et al., 1995). This is problematic as the lived experiences and cultural context of Afghan refugees largely differ from those of Israeli background. These sociocultural differences may limit the tool's accuracy, applicability, and validity in measuring the mental health symptoms of Afghan refugees. In contrast, two studies in this review employed the Afghan Symptoms Checklist, a 22-item culturally grounded instrument designed specifically to capture local beliefs and idioms of distress in Afghan populations. The Afghan Symptoms Checklist was tested in a sample of 324 Afghan adults and has demonstrated excellent reliability (Cronbach's = 0.93) and construct validity (*r* = 0.70) (Miller et al., 2006). As a result, psychological distress levels are not accurately captured in this review. Future studies should consider adopting culturally specific distress indicators to enhance the accuracy and relevance of mental health assessment in this population.

Ventevogel and Faiz (2018) have contended that the elevated prevalence rates of PTSD and depression in Afghan populations are conflated with psychosocial distress in response to chronic hardship and do not adequately capture the lived experiences of Afghans. Similarly, Alemi et al. (2023) argued that most studies employ a Western diagnostic framework and rely on self-report questionnaires that lack cultural and contextual validation. While these critiques do not diminish the reality of mental health challenges among Afghan refugees, this underscores the need to use culturally grounded, contextually relevant instruments to assess the mental health of this vulnerable population. This review did not identify any studies that explored the prevalence of anxiety or depression among resettled Afghan refugees. However, systematic reviews and studies conducted in other high-income countries found that anxiety and depression are also heightened in this population (Alemi et al., 2014; Slewa-Younan et al., 2017). Similarly, there were no studies in the North American context that examined the mental health symptoms of refugee minors. Yet, refugee children and youths are especially vulnerable and susceptible to the development of short- and long-term mental health concerns (Hazer & Gredebäck, 2023). Solberg et al. (2020) conducted a nationwide study in Sweden to evaluate the PTSD prevalence among 5,071 refugee minors from Afghanistan, Iraq, and Syria. The researchers reported that Afghan minors exhibited the highest prevalence of PTSD at 56.9%, with unaccompanied minors having increased odds of developing PTSD compared to their accompanied counterparts (Solberg et al., 2020). The UNHCR (2024b) estimates that 40% of forcibly displaced population are children under the age of 18 years. This suggests that a substantial portion of Afghan newcomers to North America are likely minors, and yet their unique experiences and healthcare needs remain critically understudied. This gap highlights the need to conduct larger studies to better understand the experiences of resettled Afghan refugees of all ages in North America.

In this review, several risk factors were identified to be associated with mental health conditions, and these include pre-migration trauma (e.g., war, financial instability, family separation and forced displacement) and post-migration stressors (e.g., unemployment, low income, discrimination, and dissonant acculturation). Demographic risk factors were female gender, older age, marital status (widowed), and being an ethnic minority (e.g., Tajik, Hazara, Nuristani, Uzbek). Female gender has consistently been identified as a significant risk factor for mental health disorders among Afghan refugee populations in the current review. This heightened vulnerability is often attributed to widespread gender-based violence, oppressive sociopolitical climate, and systemic and institutional discrimination (Alemi et al., 2023). The resurgence of the Taliban in 2021 has further severely restricted women's access to safety, freedom, education, and active participation in society (Human Rights Watch, 2024). Afghanistan has one of the highest reported rates of domestic violence globally, with 55.5% of women in a national survey of 21,234 respondents reporting experiences of physical, emotional, or sexual violence (Shinwari et al., 2022). Survivors frequently face widespread stigma from families, which is structurally embedded within the patriarchal societal structures of Afghanistan. Survivors are often silenced as domestic violence is viewed as a private affair, and women fear bringing shame or dishonour to their families (Mukerji et al., 2023). The psychological impact of gender-based violence likely compounds the burden of war, forced displacement, and economic instability, contributing to elevated rates of PTSD, increased suicide attempts, and functional impairment due to mental illness among Afghan women (Alemi et al., 2023; Kovess-Masfety et al., 2021). Evidence suggests that Afghan women may continue to follow traditional gender norms and ideologies after resettling in host countries (Afrouz et al., 2023; Siddiq et al., 2023).

While the exploration of psychosocial interventions is beyond the scope of this review, a growing body of research is examining the effectiveness of mental health interventions. There is a wide range of culturally adapted approaches that researchers have tested with promising results, including cognitive-behavioural therapy, value-based counselling, emotion-regulation programs, and multi-modal interventions (Alemi et al., 2023; Hosseini et al., 2024). Notably, there is limited research exploring the efficacy of these interventions in developed nations.

No studies specifically explored the barriers, facilitators, and practices of mental healthcare utilization among Afghan refugees. Two studies in this review examined families’ experiences of accessing pediatric mental health services. Parents expressed trust in the healthcare system and described building strong support networks (Rosenberg et al., 2022). A previous systematic review suggested that mental health and psychosocial support services were underutilized among refugees and asylum seekers (Satinsky et al., 2019). Similarly, another study found that more than half of resettled Afghan refugees with clinically probable PTSD did not seek professional health services, and only 4.6% of the total sample accessed specialized trauma and torture mental health services (Slewa-Younan et al., 2017). This underutilization of professional mental health services among refugees may be explained by potential barriers, such as language difficulties, limited awareness of available services, inaccessibility of services, stigma, cultural perceptions, and reluctance to seek formal care (Satinsky et al., 2019).

This review only identified one article that explored the health status of children of Afghan refugees. This lack of data is concerning, as the health profiles and medical needs of Afghan refugees markedly differ from the local population. Existing evidence in Europe found that the most commonly reported health concerns among Afghan refugees in Europe include infectious diseases (e.g., tuberculosis and intestinal parasitic infections), non-communicable conditions (e.g., anemia, malnutrition, and cardiovascular diseases), and mental health conditions (e.g., depression, PTSD) (Matsangos et al., 2022). These findings further highlight the differences in health conditions between Afghan refugees and host populations. Further research on refugee health profiles will equip healthcare providers with the knowledge to effectively identify, manage, and support the unique medical and psychosocial needs of this population.

The healthcare experiences documented in this review were largely positive, with many Afghan refugees expressing appreciation for the care and resources that they received (Rosenberg et al., 2022; Worabo et al., 2024). Several challenges were also identified, including language barriers, health illiteracy, lower educational attainment, transportation difficulties, and financial constraints. These challenges are organized into three domains: individual-level factors such as language difficulties, low health literacy, employment or financial constraints, unfamiliarity of healthcare services, previous negative experiences or distrust of the system; sociocultural barriers, stigma, discrimination; and systemic issues such as limited transportation, location or inaccessibility of services, funding constraints, decreased availabilities of interpreters or health services, and lack of cultural competence among healthcare providers, and (Coumans & Wark, 2024; Parajuli & Horey, 2020). Coumans and Wark (2024) further emphasized that community integration through affordable housing, employment, language training, and health information adaptation may promote resettlement and enhance healthcare access.

### Limitations

This review has several limitations. The number of eligible studies was small (*n* = 17), which restricts the breadth of available evidence. The review focused exclusively on the North American context, which may have excluded potentially relevant findings from other high-income countries, such as, Australia and Europe. This geographic constraint limits the global applicability of the findings, particularly as refugee experiences may vary significantly depending on immigration policies, societal attitudes, and settlement infrastructures. Even within North America, the evidence heavily skewed towards U.S. experiences with relatively few Canadian research and zero studies conducted in Mexico. Most included studies examined mental health, leaving important areas such as employment, acculturation, language acquisition, and broader health needs underexplored. Study samples were heterogeneous and composed of mostly adults, offering limited insight into the distinct experiences of Afghan children and youth.

## Conclusion

Four decades of conflict and instability have led to the forcible displacement of millions of Afghan refugees worldwide, resulting in one of the largest and most prolonged refugee crises in recent history. As Canada continues to position itself as a global leader in refugee resettlement, understanding the unique needs and experiences of Afghan refugees is critical. This scoping review synthesized the available evidence on Afghan refugee settlement experiences in the North American context, addressing four key objectives: to explore their post-settlement experiences, identify the facilitators and barriers they encounter, and map existing gaps in the literature. Across the included studies, mental health emerged as the most extensively researched area. Afghan refugees consistently reported high levels of mental health symptoms, which is the result of the cumulative effects of pre- and post-migration traumas. While healthcare experiences were generally reported as positive, Afghan refugees continue to face barriers to accessing healthcare. Significant research gaps remain on topics such as health conditions across age groups, effectiveness of psychosocial interventions, overall health statuses of resettled refugees, and broader determinants of resettlement, such as food security, housing, employment, and social integration.
